# 3D printed ceramics as solid supports for enzyme immobilization: an automated DoE approach for applications in continuous flow

**DOI:** 10.1007/s41981-021-00163-4

**Published:** 2021-04-29

**Authors:** Alessia Valotta, Manuel C. Maier, Sebastian Soritz, Magdalena Pauritsch, Michael Koenig, Dominik Brouczek, Martin Schwentenwein, Heidrun Gruber-Woelfler

**Affiliations:** 1 Institute of Process and Particle Engineering, Graz University of Technology, Graz, Austria; 2 CATalytic mechanisms and AppLications of OXidoreductases (CATALOX), Graz, Austria; 3 Center for Continuous Flow Synthesis and Processing (CCFLOW), Research Center Pharmaceutical Engineering GmbH (RCPE), Graz, Austria; 4 Lithoz GmbH, Vienna, Austria

**Keywords:** 3D printing, Biocatalysis, Automation, Continuous flow, DoE.

## Abstract

In recent years, 3D printing has emerged in the field of chemical engineering as a powerful manufacturing technique to rapidly design and produce tailor-made reaction equipment. In fact, reactors with complex internal geometries can be easily fabricated, optimized and interchanged in order to respond to precise process needs, such as improved mixing and increased surface area. These advantages make them interesting especially for catalytic applications, since customized structured bed reactors can be easily produced. 3D printing applications are not limited to reactor design, it is also possible to realize functional low cost alternatives to analytical equipment that can be used to increase the level of process understanding while keeping the investment costs low. In this work, in-house designed ceramic structured inserts printed via vat photopolymerization (VPP) are presented and characterized. The flow behavior inside these inserts was determined with residence time distribution (RTD) experiments enabled by in-house designed and 3D printed inline photometric flow cells. As a proof of concept, these structured inserts were fitted in an HPLC column to serve as solid inorganic supports for the immobilization of the enzyme Phenolic acid Decarboxylase (*bs*PAD), which catalyzes the decarboxylation of cinnamic acids. The conversion of coumaric acid to vinylphenol was chosen as a model system to prove the implementation of these engineered inserts in a continuous biocatalytic application with high product yield and process stability. The setup was further automated in order to quickly identify the optimum operating conditions via a Design of Experiments (DoE) approach. The use of a systematic optimization, together with the adaptability of 3D printed equipment to the process requirements, render the presented approach highly promising for a more feasible implementation of biocatalysts in continuous industrial processes.

**Supplementary Information:**

The online version contains supplementary material available at 10.1007/s41981-021-00163-4.

**Figure:**
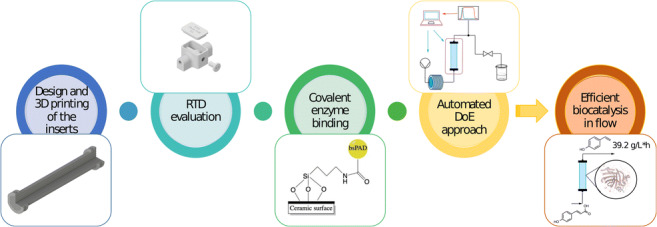
Graphical abstract

## Introduction

In recent years, the interest in 3D printing has expanded to many branches of science and engineering, due to the decreasing cost of desktop printers and the increasing choice of printing materials available [1, 2]. In the field of chemical engineering, 3D printing has been identified as a new solution to rapidly design and produce tailor-made reaction ware. This is particularly interesting for the pharmaceutical industry, since active pharmaceutical ingredients (APIs) are structurally complex and equipment flexibility is required to produce the target compounds, thereby batch reactors have traditionally been the equipment of choice [3]. Nevertheless, several pharmaceutical companies have expressed increasing interest in switching to a continuous production mode, due to the numerous advantages, such as constant product quality and easier optimization of energy and resources [4, 5]. However the low flexibility of continuous equipment still impedes the implementation of continuous processes into the pharmaceutical sector [5]. 3D printing of customized reaction ware and equipment might be the solution to this issue. In fact, this manufacturing technique allows to implement non-conventional and complex reactor geometries, whose parameters can be quickly optimized based on experimental data by iterative design modifications [6]. Also, 3D printing in chemical synthesis is not limited to reactor design, it is very often used to produce customized pieces of equipment, e.g. common laboratory hardware or sensor analytic applications [1, 7]. Different additive manufacturing techniques are available, depending on the specific purpose, but the main working principle is similar: the object, designed with computer aided design (CAD) software is sliced in a slicing software in a number of cross sections. The sliced object is then uploaded to a printer, which shapes the object by selectively stacking these layers above one another [2]. How these layers are built depends on the printing principle: in this work, we utilized vat photopolymerization (VPP) and material extrusion (ME). VPP, also known as stereolithography (SLA), generates a 3D object by selective layer-by-layer solidification of a liquid photopolymer resin using a UV light source. VPP is renowned for its high resolution of the printed parts and for the high quality of surface finish [8, 9]. Moreover, this technique is widely used for printing materials with excellent mechanical properties, such as ceramics, by suspending solid particles within the resin mixtures. ME instead is an extrusion-based technique, in which a thermoplastic polymer is melted and extruded through a hot nozzle. The polymer is then deposited onto a building plate through the nozzle in a layer-by-layer fashion, thereby creating an object by stacking different slices on top of each other [8]. After the layer deposition, the polymer hardens on cooling, which might cause adjacent layers to not bind properly. In general, this results in low mechanical stability of the printed parts along the z-direction [10] and possible fluid leakage during application. However, the mechanical properties can be improved in the post-processing phase via steps of drying, heating, or sintering [11]. The resolution for ME printed parts is lower, but this technique is still widely used due to its high versatility and low costs [11].

As mentioned above, 3D printed reactors are widely used in flow chemistry, and many reviews can be found on this topic [1, 10, 12–14]. Nevertheless, the use of 3D printing to realize supports and internals for structured reactors is more recent and still limited to few applications [15, 16]. In industry, packed bed reactors filled with heterogenous catalyst powder are still preferred due to the facile utilization and the high surface area available for mass transfer. However, they pose some operational difficulties, such as high pressure drops and flow maldistributions, with consequent channeling and decrease in the overall process performance [8]. Therefore, structured catalysts and packing materials have been developed to overcome these issues. Characterized by a flexible design, they can be implemented into commercially available columns and reactors. Furthermore, they can be easily interchanged, which makes them particularly interesting as catalyst support. The catalyst can be immobilized on the solid matrix as a washcoat or incorporated in the chemical structure of the support material [1, 8]. Moreover, by using additive manufacturing technologies, it is possible to design and engineer the support structures to perfectly meet the reaction requirements, such as fast mixing, improved heat transfer and fluid distribution (e.g. reduced channeling) compared to randomly and particle-based packed beds [17]. In addition, rapid optimization of the support structure is straightforward and can be achieved by iteratively improving the design based on the collected experimental data. Finally, catalytic supports are much easier to handle and to fill into a reactor compared to heterogeneous catalyst powders.

In this work, structured ceramic supports printed via vat photopolymerization and their use for catalytic applications are presented. In order to describe the microfluidic behavior inside the supports, low cost flow cells were designed to easily perform residence time distribution (RTD) experiments. The RTD setup includes two 3D printed flow cells, common laser beams as light source, and an Arduino microcontroller to monitor the change in absorbance when a dye is injected into a microfluidic device. The setup was designed to be an efficient and low cost alternative to flow photometric equipment available on the market and reflects the power of 3D printing to design customized analytical tools without having to purchase expensive and patented equipment [18, 19]. By combining 3D printed parts with low-cost microcontrollers and electronics, it is possible to extend the scope of in-house designed laboratory equipment to more complex applications, such as inline and real-time monitoring of defined process parameters [19–21] For rapid prototyping purposes, Arduino microcontrollers are the most known and commonly used, due to the many advantages they offer. Firstly, they are cheap and can be coupled with a wide range of sensors and devices. Secondly, the software and the board are user friendly, which makes it easier for scientists with little programming background to create their own prototype. Finally, Arduino is an open source project with a wide community of users, which increases the possibility of sharing ideas and prototypes among labs and researchers [20].

As an application-oriented proof of concept, in this work the model enzyme Phenolic Acid Decarboxylase from *Bacillus Subtilis* (bsPAD) was covalently immobilized onto 3D printed solid supports and tested for the decarboxylation of coumaric acid in a continuous flow setup. The system was chosen due to the considerable industrial and economical interest in biocatalysis and the ongoing efforts to increase the feasibility of enzymes by finding innovative and optimal supports for their immobilization [22]. In fact, biocatalysis has emerged as a green and highly efficient alternative to metal-based catalysis for the manufacturing of APIs and fine chemicals [23]. Enzymes are natural and environmentally friendly catalysts, showing high activity in mild conditions (low temperatures and water-based solvents) as well as high substrate specificity. Moreover, due to past advances in protein expression and purification, the price of enzymes has decreased strongly over the last years, making enzymes more economically feasible even for large scale processes [24]. However, the limited long term stability and reusability of enzymes make it very challenging for this technology to be competitive on an industrial level [22, 23]. Hence, enzyme immobilization has been proposed as a very efficient solution to overcome these issues [22]. Various methods for immobilization are available and differ in the mechanism of protein attachment, such as affinity bonding, physical adsorption, covalent bonding and encapsulation. Each of these methods has its advantage and drawbacks, and the choice greatly depends on the specific application [23–26]. Covalent binding is commonly used when enzyme immobilization onto a solid support material is targeted, as the support surface can be easily modified and functional groups for enzyme attachment are facile to introduce. This technique allows to preserve the enzymatic activity for a longer period of time, reuse of the biocatalyst as well as easy enzyme separation from the reaction mixture [26]. Several organic and inorganic materials have been used as matrix for covalent enzyme attachment including polymers commonly used in additive manufacturing [23, 24, 27]. This has opened up the possibility to 3D print bioreactors or carriers made from commercially available resins, allowing direct immobilization of the enzymes onto the structures’ surface and enabling high performance and recyclability [28–30]. Regarding inorganic materials, enzymes have been very often covalently attached onto silica or ceramic particles and/or monoliths [30–33]. However, to the best of our knowledge, this is the first report of enzyme immobilization onto 3D printed ceramic supports.

**Scheme 1:**
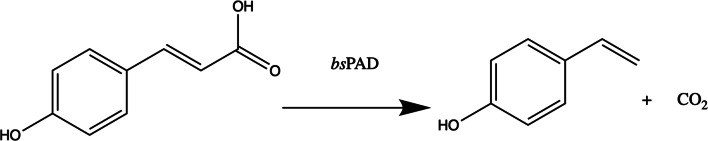
Reaction scheme for the enzymatic decarboxylation of coumaric acid to vinylphenol catalyzed by bsPAD

In this work, we examined the continuous transformation of *para*-coumaric acid into vinylphenol catalyzed by *bs*PAD (Scheme 1) covalently immobilized onto our 3D printed inserts. Since this enzymatic reaction had already been studied previously by our group using an encapsulated biocatalyst in continuous flow [34], in this work we decided to utilize a ceramic support material and focus on determining the influence of different internal geometries and process parameters on the reaction outcome. Ceramics was preferred to standard 3D printing resins as a support material since it is chemically inert and does not pose any risks of inactivation to the enzyme in use. In the case of *bs*PAD, the choice of using ceramics resulted in a great compatibility of the enzyme to the carrier material, as demonstrated by the long-term activity and stability achieved. Moreover, ceramics is less brittle than standard resins [1], which makes it easier for an insert to be tightly packed in a column without being crushed. Alumina is also cheap and widely available, therefore it is possible to easily produce objects with great mechanical and chemical properties at an affordable cost.

In order to decrease the time needed for screening the different selected parameters and increase the level of process understanding, an automated process setup was realized, as shown in Scheme 3. The possibility of process automation is one of the many advantages of continuous manufacturing, as it allows for rapid and controlled screening of process parameters [35–37]. On lab scale, automated reaction platforms are enabled by using standard hardware-connectivity to remotely control the equipment, and online analytics to monitor the process parameters in real time [36, 38]. In combination with computer algorithms, it is possible to perform experiments automatically by controlling the equipment, data acquisition, and performing process optimization based on the recorded data [36, 38]. Multidimensional systematic optimization strategies, such as Design of Experiments (DoE), can be easily implemented in such automated platforms and are generally preferred to the one-variable-at-a-time (OVAT) approach [39–41]. In the DoE approach, process parameters are first ‘screened’ to identify the critical factors influencing the reaction outcome (e.g. for a chemical reaction: yield, purity, cost, etc.). Then, an ‘optimization’ step is carried out to determine the best settings for the individual variables [40]. DoE also reveals more information on the interactions between the process parameters and their effect on the outcome of the reaction, and allows to save time and materials by minimizing the total number of experiments needed [39].

In this work, we have applied a fractional factorial Central Composite Design (CCD) [42, 43] for the DoE study to investigate several process parameters and gather as much information as possible on the *bs*PAD catalyzed reaction using our structured inserts. CCD was also used to define the optimal carrier and combination of process parameters. The fractional factorial CCD approach was preferred over the full factorial approach and similar approaches such as the Box-Benkhen design due to the lower number of experiments needed per iteration [35, 44].

Overall, the goals of this work are to show the power of 3D printing for designing flexible and easily interchangeable structured inserts, as well as for realizing powerful, yet low cost inline analytical tools to increase process understanding. To achieve these goals, two different types of structured inserts were designed, based on two different internal geometries. To determine the flow pattern inside the inserts, RTD experiments enabled by our in-house designed and printed inline photometric flow cells were used. Then the decarboxylation of coumaric acid catalyzed by *bs*PAD was chosen as a model system to prove that it is possible to use these engineered inserts as structured bed reactors in a continuous biocatalytic application, with great results in terms of productivity and process stability. By automating this setup, it was also feasible to quickly identify the optimum operating conditions for the decarboxylation reaction, thereby exploiting the advantages of flow chemistry to the fullest.

## Results and discussion

### Design and fabrication of the 3D printed inserts

**Fig. 1:**
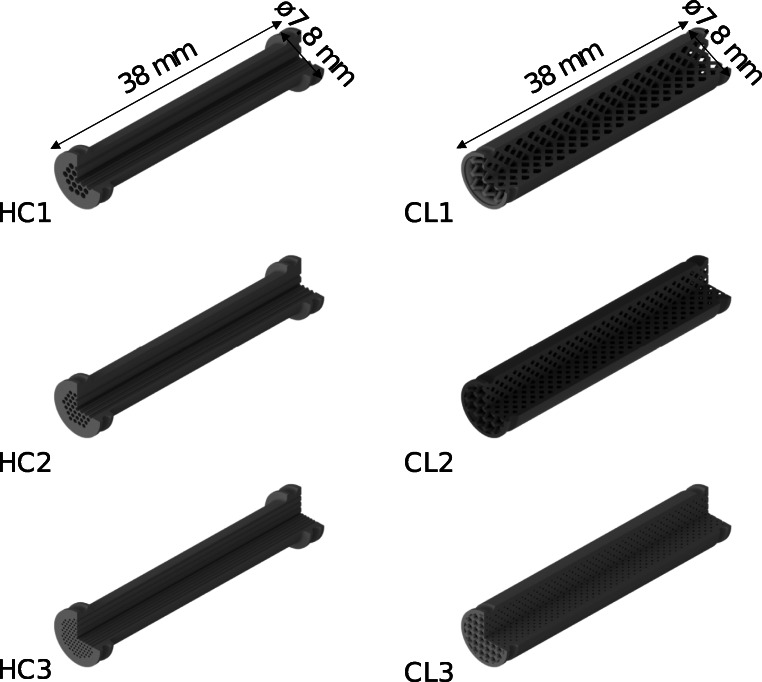
CAD drawings of the designed and 3D-printed ceramic inserts. Straight honeycomb like structures (HC, left) and cubic lattice (CL, right). From top to bottom, the hydraulic diameter of a respective structure decreases, while the internal surface to volume ratio increases

The supports used for immobilization of the enzyme were designed using Autodesk®’s CAD software Inventor® and printed at Lithoz GmbH. Two different internal structure types were realized, by following a similar approach from literature [17]. The first one consists of a honeycomb-like structure (HC) comprising internal straight channels of hexagonal cross sections (Fig. 1 left). This design was chosen to achieve fluid distribution along the channels, depending on the individual channel backpressure, whereby no mixing can take place between them. The purpose of this design was to approach a plug flow behavior while reducing the extent of backmixing. However the individual backpressure of the channels can cause the fluid to not distribute evenly among the channels and might affect a structure’s performance. The second structure features a cubic lattice (CL) design that is repeated along the length of the support (Fig. 1 right). In this way, radial mixing is introduced, while increasing the available surface area. Both designs are equipped with an outer shell and an O-ring on both sides to fix the support tightly when inserted into a column, thereby preventing bypassing of the fluid. The outer diameter of all supports was set to 7.8 mm at the O-ring section and kept at this diameter for the lattice structure. The O-ring prevented adding channels close to the outer radius of the honeycomb as it would have blocked them. As a consequence, the unused mass in the middle of this design needed to be reduced as it was likely to produce cracks during post-processing. A final diameter of 5.8 mm was chosen for the middle section. Both designs feature a length of 39.8 mm in order to fit perfectly into commercially available empty HPLC columns (ID 8 mm, height 40 mm). All designs were replicated and printed with 3 different channel sizes. The geometrical characteristics are summarized in Table 1.

The structures were printed using a lithography-based ceramic manufacturing technique. The printer used was a CeraFab 7500 and the printing setup is shown in the Supporting Information (SI, Figure S3). It features a transparent vat into which the slurry, made of a mixture of monomer, photoinitiator and alumina, is automatically dispersed and spread. The movable building platform is immersed into the slurry, which is then selectively exposed to visible light from below the vat. The layer image is created via a digital micro-mirror device (DMD) coupled with an advanced projection system. By repeating this process, a three-dimensional green part can be generated layer-by-layer. A support structure was used for each column to avoid over polymerization in the channels. More information on the building parameters can be found in the SI. After printing, the parts were cleaned with pressurized air and a cleaning solvent. Then, thermal postprocessing was carried out by placing all parts in a furnace and first applying a preconditioning cycle at 120 °C, followed by debinding and sintering at 1500 °C for 2 h.

**Table 1:** Summary of the geometrical characteristics of the designed inserts

Type of insert	Number	Hydraulic diameter [mm]	Internal volume [mm^3^]	Internal area [mm^2^]	Area/Volume [mm^2^/mm³]
	1	0.757	351.71	1857.31	5.3
Honeycomb (HC)	2	0.521	314.86	2416.17	7.7
	3	0.263	234.65	3572.21	15.2
	1	1.564	1044.78	2671.38	2.6
Cubic lattice (CL)	2	0.930	842.98	3627.25	4.3
	3	0.433	467.12	4319.53	9.2

The internal volume and area were calculated in Autodesk Inventor

### Characterization of the inserts– determination of the mixing behavior/flow pattern

**Scheme 2:**
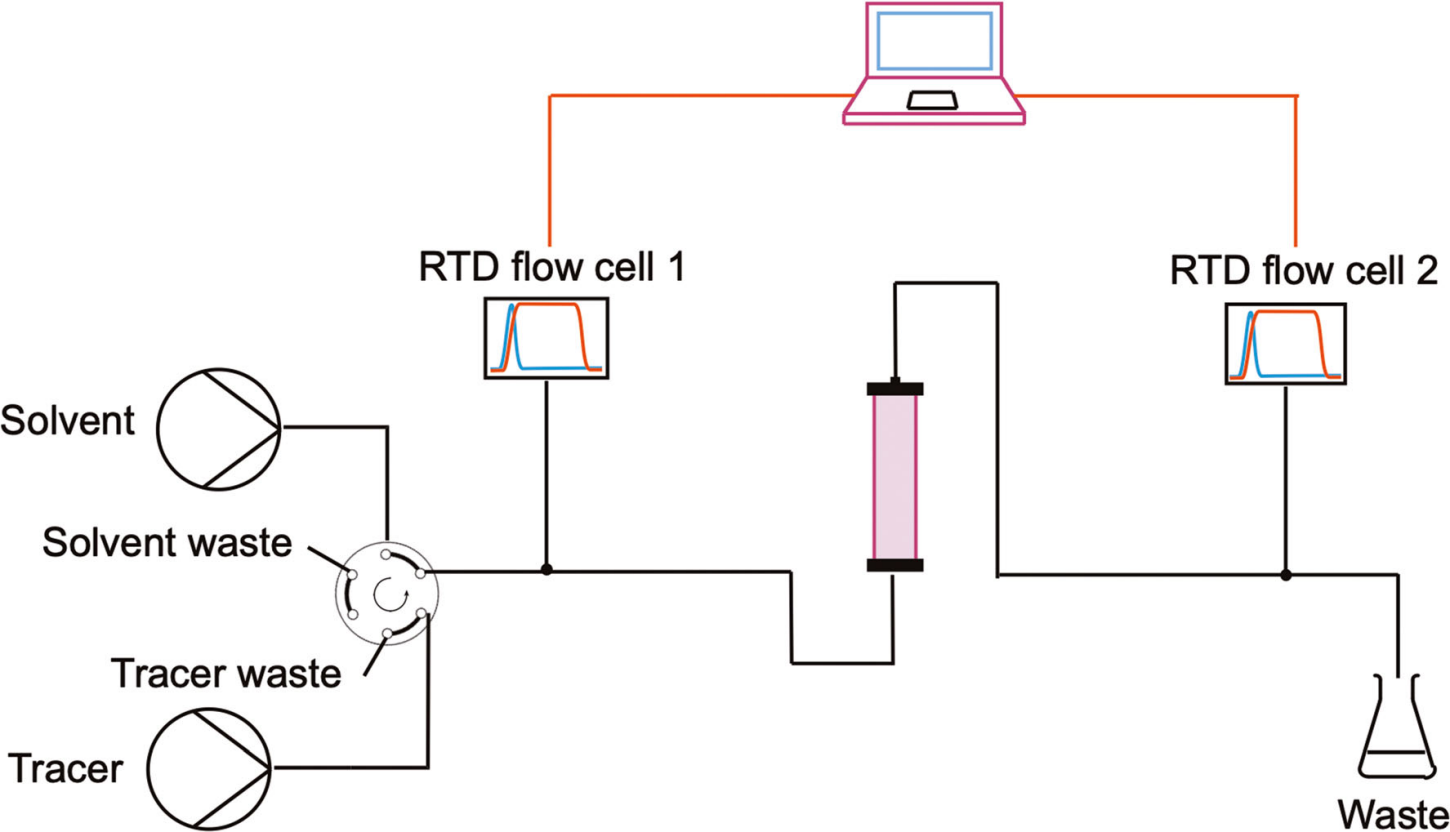
Setup for the evaluation of the Residence Time Distribution (RTD) and thus the flow patterns inside the designed structured inserts

In order to assess the flow pattern inside of the columns packed with designed inserts, experiments were carried out to determine the residence time distribution (RTD) using the step input principle, as reported in literature [45]. The detailed procedure of the measurements is provided in the SI, and the setup is presented in Scheme 2. Two syringe pumps were used, one filled with ethanol and one with a mixture of ethanol and methylene blue as tracer. Both were connected to a six-way-valve, controlling the flow through the structured insert, which was incorporated in a HPLC column. Two flow cells were implemented in the system, one at the inlet and one at the outlet of the column. With this setup any sources of disturbances and deviations occurring not only inside of the column, but also at its inlet are taken into account. For example, using a manually switched valve to inject the tracer can lead to disturbances in the evaluation of the step signal, as the time for injection is not univocal for all experiments and the step itself is not ideal. Another issue lies in the feeding tube or capillary used to deliver the tracer to the HPLC column. If the axial dispersion in this capillary is high, this might influence the RTD, thereby falsifying the results. This effect is especially relevant for low Reynolds number ranges (as it is the case for microfluidic applications). One way often proposed in literature to compensate for deviations in the system is to record the tracer signal at the inlet and at the outlet of the device. The results are then analyzed by using the approach of the convolution integral theorem, to take into account deviations to the RTD being caused by auxiliary equipment [45, 46].

For the experiments, 3D printed flow cells mountable on the outside of transparent 1/16” capillaries were designed (see SI Figure S4). Their measurement principle is based on a light intensity measurement passed through a capillary, whereby each flow cell represents a photometer on its own. The emitted light of a red LED is absorbed within a capillary and its fluidic content and finally detected on the other side by a photo resistor. The photo resistor changes its conductivity depending on the absorbed light and allows measuring of light intensity depending voltages. This voltage is recognized by an analog to digital converter (ADS1115, 16 bit). To account for different mounting and manufacturing deviations of the electronic parts, an adjustable voltage divider was added to tune offset values. Buttons for LED control and reference points are added as well, to take the needed light and dark reference values. LEDs and photo resistors are powered by a micro controller (Arduino Nano), which reads the measured voltages of the ADS1115 and provides measured data to a PC via a serial communication. Beside the LED and photo resistors, all other electronic parts were soldered on a printed circuit board (PCB) to increase measurement stability. With the developed set-up, a low cost possibility to measure RTDs is presented.

The experimental procedure started by flushing the column with pure solvent and saving a light and dark reference spectrum. Then, the tracer solution was injected into the column after defined time intervals by manually switching the six-way-valve to the inject position. The change in absorbance was recorded at both the inlet and the outlet of the column and converted into a digital signal via an Arduino Nano and logged into a serial terminal program (Tera Term) on a PC. When the absorbance of the tracer reached a stable value, a step down was induced by switching the six-way-valve back to the load position. Different flowrates (2, 1, 0.5, 0.2, 0.1, 0.05 mL/min) were tested in order to evaluate how the flow pattern changes with varying Reynolds number. For each experiment, the results were exported in a .log file and imported in Microsoft Excel to evaluate the cumulative and exit age distributions. In order to quantify the extent of axial dispersion and backmixing into the devices, the Bodenstein number (*Bo*) was determined for each insert at every investigated flowrate. This dimensionless number is defined as the ratio of the convective mass transfer over axial dispersion:

$$Bo=\frac{u\bullet {L}_{char}}{{D}_{ax}}$$

where $u$ is the flow velocity, ${L}_{char}$ is the characteristic length of the device and ${D}_{ax}$is the axial dispersion coefficient. *Bo* can be used to estimate how close the fluid behavior inside of a reactor is to an ideal reactor model: for *Bo* > 100, the fluid pattern inside the investigated reactor approaches that of a plug flow reactor (PFR), while for *Bo* < 100, the behavior approaches that of a CSTR [45]. Different methods have been reported to calculate this number from the RTD curves, based on different assumptions on the boundary conditions of the system. In this work, the open-open model was chosen, as it assumes that the flow is dispersed along the length of the whole column and it also provides an analytical solution to calculate *Bo* solely from the variance of the RTD curve [47]. Moreover, since it was possible to place two flow cells at both the inlet and outlet of the columns, the calculated variance and mean residence time for each insert could be corrected by taking into account the influence of the inlet capillary on the output response. For this purpose, the additivity approach described by Levenspiel [45] was used.

**Fig. 2:**
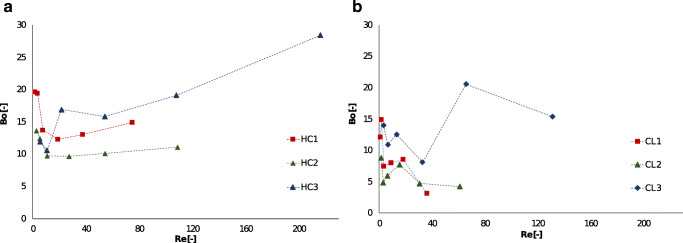
Results of the RTD experiments for the honeycomb (**a**) and the cubic lattice (**b**) structured inserts

The results for the RTD experiments are summarized in Fig. 2, by plotting the calculated *Bo* versus the Reynolds number (*Re*) for each measured point. *Re* was calculated by identifying a hydraulic diameter ${d}_{H}$ (as reported in Table 1) for each structured insert, as it allows for the comparison among different internal structures. The ${d}_{H}$ was calculated as follows:

$${d}_{H}=\frac{4\cdot A}{P}$$

where A is the cross sectional area of the device and P is the wetted perimeter, which were both estimated with the aid of Autodesk Inventor®. The *Re* was then calculated as :

$$Re=\frac{\rho \cdot u\cdot {d}_{H}}{\mu }$$

Where ρ and µ are respectively the density and the dynamic viscosity of the fluid flowing through the channel.

The results show that for all the designed inserts in the investigated flowrate region, *Bo* was significantly below 100, indicating high backmixing and axial dispersion inside the inserts. Considering the results for the honeycomb (HC) structured inserts (Fig. 2a), it can be seen that the average *Bo* was the highest for HC3 and it was rising with the flowrate. Since the superficial velocity inside a channel is increasing with decreasing inner diameter, therefore the *Re* is higher for HC3 and the flow is more chaotic, resulting in a lower extent of backmixing. Axial dispersion is instead higher in the case of HC2 and HC1: in both inserts the change in *Bo* with the flowrate was very limited, indicating that the insert was approaching CSTR behavior in the whole investigated region. Also, the values of *Bo* seemed to increase at lower *Re* for these inserts, especially for HC1, for which *Bo* was higher than the values achieved by HC3 at *Re* below 7. This trend can be explained by the fact that syringe pumps have a more pulsating and irregular behavior in this range. Another possibility is that at lower flowrates flow maldistributions might occur, because the connector that ties the capillary to the HPLC column has a small inlet in the middle, therefore the fluid might not distribute to the outer channels and flow mainly through the middle ones. Maldistributions might be more relevant for HC3 and HC2 due to the lower channel diameter and the higher number of channels, resulting in the fluid to not be distributed equally among all channels and therefore decreasing the mixing efficiency. Since HC1 has bigger channels, it is easier for the fluid to be pumped uniformly in all channels, even for low *Re*.

The trend instead changes for the square lattice structured inserts CL 1–3. As visible in Fig. 2b, no trend was recorded for *Bo* with increasing flowrate, instead it changed around an average value. A slight increase in *Bo* could only be detected for CL3, which was expected since it has the smallest hydraulic diameter. In fact, the higher density of lattice elements present inside CL3 promotes the formation of secondary flow structures, thereby increasing the extent of convective mixing. For CL1 and CL2 there was almost no difference in *Bo* for *Re* > 5. However, below that, CL1 seemed to have a similar behavior to CL3. As for the HC inserts, this inversion in the trend could be arising from fluctuations in the pumps, or it could be due to flow maldistributions inside the CL3 insert.

In general, for both types of inserts it can be expected that using higher flowrates improves mixing and promotes PFR behavior, however since higher flowrates were not interesting for the chosen application (as they result in too low residence time for the selected reaction), the investigated range was not enlarged any further.

### Decarboxylation reaction – choice of the solvent

Phenolic acid decarboxylase (PAD) is an enzyme belonging to the family of cofactor-free decarboxylase. It catalyzes the removal of carbon dioxide from hydroxycinnamic acids, such as coumaric acid, to give hydroxystyrene products that are important API precursors [48]. Even though PAD is very active towards this reaction, a limit to the industrial application is the low solubility of phenolic acids in water-based solvents. Therefore, alternative solvent systems have been proposed, in order to maintain the high activity of PAD and increasing the space-time -yield (STY) of the decarboxylation [48]. Deep Eutectic Solvents (DES) have been suggested as an alternative to ionic liquids, due to the lower toxicity and the greater uptake of the CO_2_ released during the decarboxylation [34]. They consist of a mixture of primarily quaternary ammonium salts (e.g. choline chloride) with hydrogen-bond-donors (HBD) (such as glycerol). The heating and mixing of this solution disrupt the crystalline structure of the ammonium salt, generating a viscous liquid with a lower melting point than the starting materials [34, 49].

The DES mixture of choline chloride and glycerol in a 1:2 molar ratio, diluted 1:1 (v/v) with phosphate buffer at pH 6 was identified in previous works [34, 48] as an efficient solvent for the decarboxylation, both in terms of CO_2_ absorption and high substrate solubility, since the properties of the DES are still relevant at this dilution ratio. Therefore, this solvent mixture was kept also in this work to investigate the 3D printed inserts as catalytic supports for the *bs*PAD enzyme.

The decarboxylation of coumaric acid to vinylphenol was performed first in batch for the preliminary immobilization tests (see the SI) in order to identify an optimum immobilization procedure. Finally, automated flow experiments were carried out following the procedure explained in the dedicated paragraph.

### Enzyme immobilization

Covalent binding was chosen as an immobilization strategy, since it has been widely used as a method for protein attachment on ceramic supports [50–53]. Two different methods (1 and 2) were selected and first tested in batch by using the particles obtained from grinding the ceramic objects that failed during printing. All steps for both methods are described in detail in the SI. Method 1 was taken and adapted with slight modifications from literature [50]. It involved a first step of surface activation by acid treatment, followed by a silanization step with 3-aminopropyltriethoxysilane (APTES) to introduce amino groups onto the surface, which are functionalized with glutaraldehyde. This compound comprises two reactive aldehyde groups and it is used to link two amine functionalities, one on the surface of the support and one on the enzyme itself, by forming amide bonds [54]. This method has the advantage that it ensures stable protein attachment. However, glutaraldehyde is toxic and might even deactivate the enzyme [55]. Therefore, a different immobilization procedure (method 2) was chosen, also slightly modified from literature [52, 53]. In this case, initial surface modification steps until silanization are the same, but instead of having a surface functionalization step, the enzyme was directly immobilized onto the support by introducing the less harmful linkers *N*-hydroxysuccinimide (NHS) and *N*-(3-dimethylaminopropyl)-*N*′-ethylcarbodiimide hydrochloride (EDC) into the enzyme solution. These compounds activate the available carboxylic groups on the amino acids, which undergo bond formation with the amine groups on the carriers [56]. Preliminary batch tests showed that the latter method is preferred in terms of immobilization yield and long-term stability of the enzyme activity (see SI). Therefore, this method was chosen to immobilize *bs*PAD on the 3D printed inserts for the purpose of performing the flow experiments.

The immobilization of *bs*PAD on the structured inserts was carried out in flow, as shown in the SI. This allowed to avoid material handling in between the different immobilization steps and reduced the overall time needed for the procedure. In order to estimate the amount of enzyme immobilized on the supports, the immobilization yield (${Y}_{i}$) was determined via taking 1 mL samples from the enzyme solution before and after immobilization and performing the activity assays in batch as described in the SI. By determining the activity of the solution before and after immobilization, it was possible to calculate ${Y}_{i}$ as:

$${Y}_{i}=\frac{{A}_{b}-{A}_{a}}{{A}_{b}}*100$$

Where ${A}_{b}$ is the activity before and ${A}_{a}$the activity after immobilization. The activity of *bs*PAD was defined as the amount of µmoles of coumaric acid being consumed per minute, taking the first 30 min of reaction for the calculation, as in this region the conversion of coumaric acid was linear. The calculation was carried out as follows:

$$U=\frac{\left({C}_{CA,0}-{C}_{CA,30}\right)*{V}_{assay}}{t}$$

Where ${C}_{CA,0}$ and ${C}_{CA,30}$ are the concentration of coumaric acid at the start and after 30 min into the reaction (given in µmol/L, determined by HPLC),${V}_{assay}$ is the total reaction volume and t is 30 min. The activity is therefore is given in units U [µmol/min].

The theoretical activity of the enzyme immobilized on each insert was calculated by assuming that this is equal to the difference between the activity in the starting immobilization solution and the one measured at the end of the immobilization. This value was only used as an estimation, mainly as it was not possible to access the small internal channels in order to determine the exact concentration of the bound enzyme via standard assays (e.g. protein labelling with fluorescent molecules [57]). Also it does not take into account whether the enzyme undergoes conformational changes upon covalent binding to the carrier, which might affect its structural properties compared to the free enzyme [23, 25].

The exact surface area of each uncoated insert was determined via Brunauer–Emmett–Teller (BET) measurement, as described in the SI. The results are summarized in Table 2. Only insert types 1 and 3 were tested in the continuous setup, as the inserts with the middle channel size did not show to be interesting both in terms of their fluidic behavior (as shown from the RTD results) nor available surface area (as shown from the BET results). The calculated values for ${Y}_{i}$ and the estimated activity showed that a higher amount of enzyme bounded to HC3 and CL3, most probably due to the higher surface area available for the immobilization.

**Table 2:** Overview of the measure BET surface area and the achieved immobilization of bsPAD onto the structured inserts

Type of insert	Number	BET surface area [m^2^/g]	Weight of insert [g]	Immobilization yield [%]	Estimated immobilized activity [U/g]
Honeycomb (HC)	1	0.254	2.32	19	1.24
	2	0.310	2.5		
	3	0.543	2.86	58	6.02
Cubic lattice (CL)	1	0.484	1.98	14	1.09
	2	0.522	2.52		
	3	0.536	3.77	50	2.62

### Automated flow setup

**Scheme 3:**
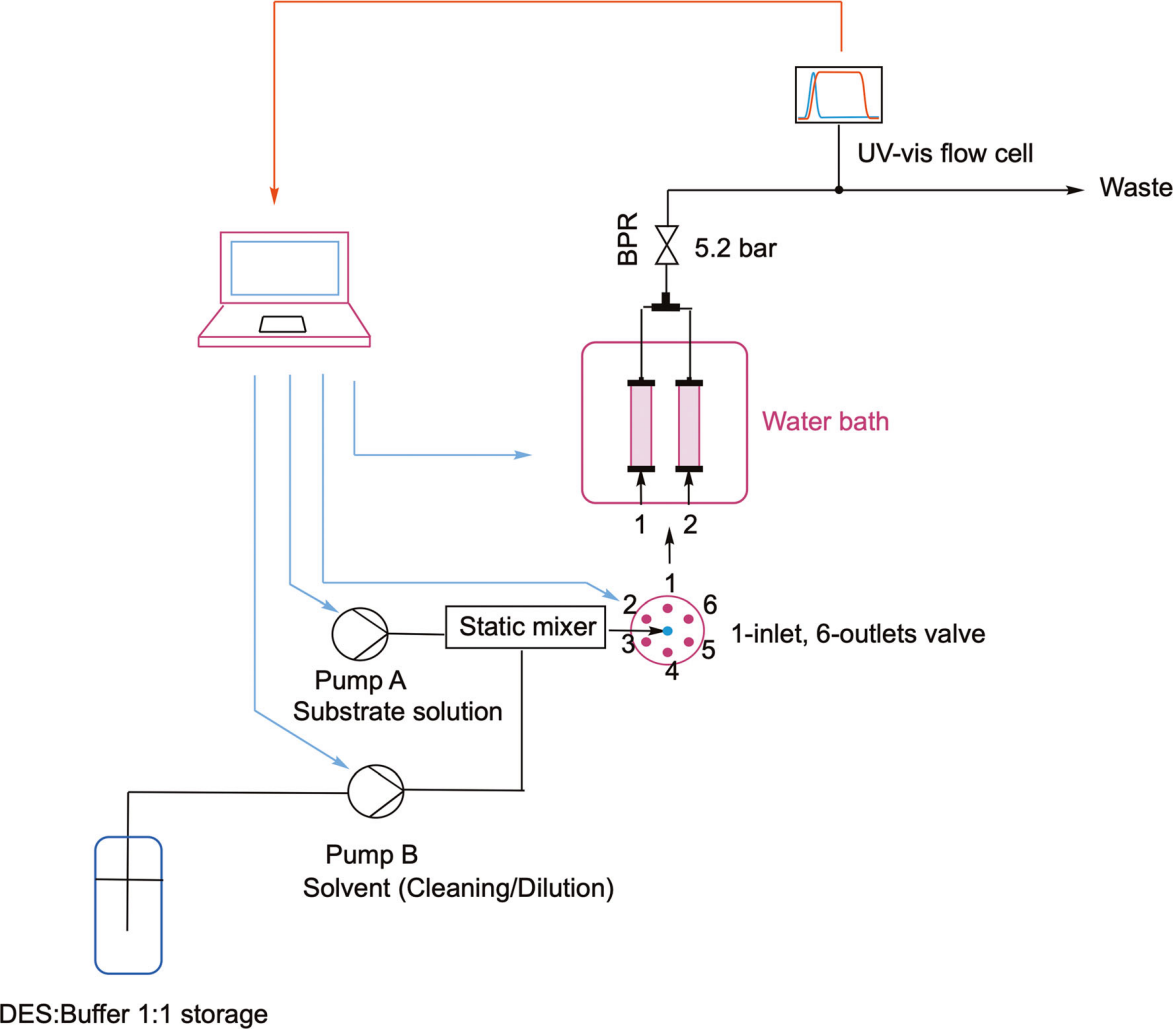
Setup for the automated screening and optimization of the decarboxylation of coumaric acid catalyzed by bsPAD immobilized onto 3D printed inserts. DES = Deep eutectic solvent (ChCl/Gly 1:2 mol)

After being coated with *bs*PAD, the inserts are ready to be used in the continuous flow setup shown in Scheme 3. The scope of this setup was to allow the automated evaluation of the different coated carriers and the effect that changing temperature, flowrate and substrate concentration have on the decarboxylation reaction, according to a DoE approach.

The structured inserts were again enclosed into HPLC columns and constituted the structured bed reactors at the heart of the process. The setup featured one HPLC pump (Knauer Azura P 4.1 S, pump B in Scheme 3) delivering solvent, and a syringe pump (Lambda VIT-FIT, pump A in Scheme 3) equipped with a 50 mL stainless steel syringe filled with a 10 mM stock solution of coumaric acid. The outlets of the pumps were then connected to a static mixer, in order to provide a feed stream with uniform concentration before it reaches the column inlet. As static mixer, a 3D printed stainless steel microfluidic device was used, which was designed in our group and characterized in a previous work [58]. This chosen device was the so-called AP04 with an internal diameter of 0.6 mm, into which the solvent and the substrate solution were mixed according to a split-and-recombine principle. The AP04 was chosen as static mixer since it was proved in a previous publication [58] that it provides reasonably good degrees of mixing while remaining a compact device. The feed was then pumped into one of the columns, each connected to a six-way-valve (Knauer Azura VU 4.1), which dictates the column into which the feed is pumped to. Two inserts of the same type (e.g. HC1 and HC3, or CL1 and CL3) were evaluated at a time, by being fitted in HPLC columns and then connected to ports 1 and 2 of the six-way-valve. The columns were immersed in a temperature-controlled water bath to keep the temperature at the desired value. The pumps, six-way-valve and thermostat were connected to a PC and controlled via a Python-based script. In this way, the automated control and change of temperature, flow rate and substrate concentration for each experiment were facilitated.

Before starting the automated experimental sequence, the whole setup including the columns was flushed for at least 1 h in order to remove loosely bound enzyme and take a blank reference for the UV-vis measurement. Then starting with column one, the DoE algorithm performed different experimental runs in order to screen the reaction space as determined by the fractional CCD method, which is described in detail in the next section. During each run, the reaction was monitored via an Avantes Micro Flow Cell with 1.5 mm path length connected to a spectrometer (AvaLight-DS-DUV equipped with a deuterium lamp and AvaSpec-ULS2048 detector), allowing the real-time tracking of the rate of vinylphenol formation. The product was identified by a characteristic peak at the wavelengths between 269 and 263 nm, whereas the reagent was measured at wavelengths between 325 and 328 nm, where vinylphenol does not absorb. From this data and previously determined calibration curves, the conversion of coumaric acid and the yield of vinylphenol were calculated. After having performed all experimental runs on the first column, the algorithm prompted the valve to switch to the second column, and the same optimization algorithm was repeated. Once both inserts had been evaluated, the columns were removed from the thermostat and the inserts were exchanged manually to the other design type. Then, the columns were reinstalled in the setup and the DoE evaluation was started again by following the same procedure as before.

### Design of experiments (DoE)

Fractional CCD was chosen for the DoE study due to its efficiency for screening a high number of reaction parameters using a low number of experiments. Regarding the particular enzymatic reaction studied in this work, it was highly desired to reduce the amount of experiments to not waste solvent or reagent, thereby reducing the amount of waste and containing the costs. Three factors were chosen for optimization: feed flowrate, reaction temperature and substrate concentration. These values were evaluated within a limited design space reported in Table 3. The maximization of the yield and space-time-yield (STY) of vinylphenol were chosen as the target response of the DoE. The STY was calculated according to the following equation [59], assuming that the enzyme is forming a monolayer on the inner surface of the insert:

$$STY=\frac{{c}_{prod}\bullet FR}{{V}_{i}}$$

where ${c}_{prod}$ is the concentration of product formed (in g vinylphenol/L) at steady state at the chosen experimental conditions, FR is the flowrate (in L/h) and ${V}_{i}$ is the internal volume of the reactor (in L).

**Table 3:** Boundaries of the design space investigated in the DoE experiments

Parameter	Lower limit	Upper limit
Flowrate	0.2 mL/min	2 mL/min
Temperature	25 °C	35 °C
Substrate concentration	0.25 mM	2 mM
Dilution ratio	4	40

The upper and lower limits for the flow rate were chosen considering a sufficient residence time within the column as well as concerning the structural limits of the UV-vis flow cell (it can only withstand a pressure up to 10 bar). For the temperature, the limits were set around the optimal temperature defined in a previous work [34]. In terms of the substrate concentration, the low solubility of coumaric acid in the reaction solvent and the flow cell path length were the limiting factors. The starting substrate concentration for each experiment was set in the platform by changing the flowrates of the solvent and the substrate pumps in order to achieve the desired dilution ratio. Therefore, dilution ratio was given as an input parameter to the DoE algorithm.

**Fig. 3:**
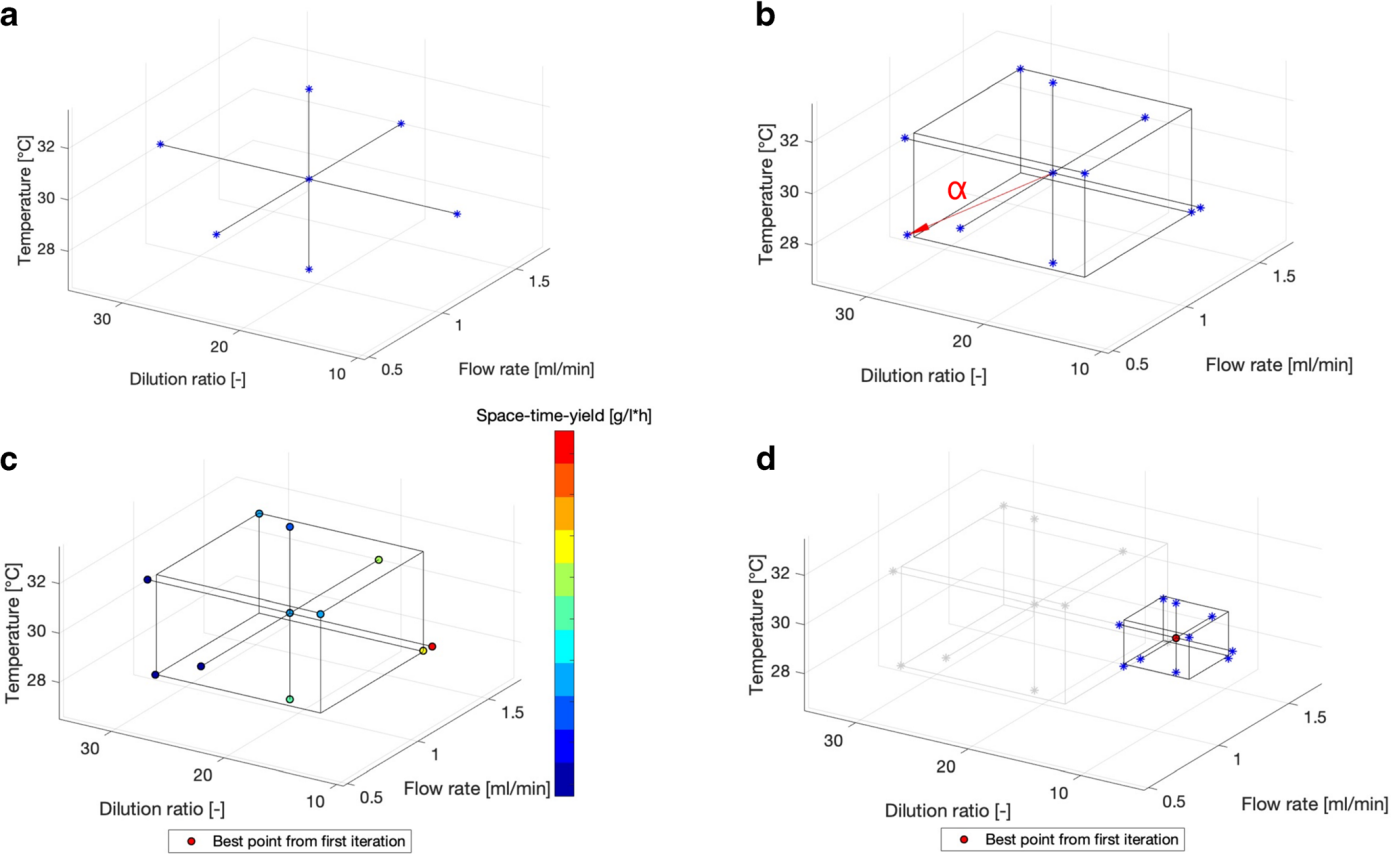
Graphical representation of the steps for the chosen Fractional Factorial CCD approach. For each structured reactor, two DoE runs were performed. First, the algorithm choses the center point of the DoE according to the boundaries of the design space given by the user. Then, the high and low levels for each factor are also identified and combined to give the axial experimental points (**a**). Since this is a CCD approach, further points are added to the experiments, which lie at the corners of a cube that has a fixed length (identified as α) and are at the same distance from the center point (**b**). However not all corner points are selected as experimental runs, but only 4, as this is a fractional design. Once all points are defined, the algorithm performs all 11 experiments automatically, and choses the best point based on the highest STY (**c**). A new iteration is then started (**d**) by choosing 10 points around the best point from the first iteration, which becomes the new center point, following the same procedure as in the first iteration but by scanning a smaller area of the design space

A scheme summarizing the steps for the chosen experimental design is shown in Fig. 3. For each 3D printed structured insert, two DoE runs were performed. In the first run, a subsection of 70 % of the total design space was screened to identify an optimum combination of parameters (each represented as a point) and narrow down the experimental space. Based on the boundaries given by the user, the algorithm defined the center point and the high and low levels for each factor, which were connected by a cross on each plane in the 3D design space (Fig. 3a) and constituted the axial experimental points, resulting in 7 experiments. Then, further points were added to the starting ones, and were chosen by the algorithm based on a spherical composite design approach [43]. According to this approach, a cube is drawn, which is centered at the center point of the design and has a side length of α. This distance is defined as the square root of the total amount of factors and was 3^1/2^ for this work. The new experimental points lie at the corners of the cube, and are therefore equally distant from the center. Since a fractional factorial design was applied, only 4 of the corner points were added to the total experiments, resulting in a final amount of 11 experimental runs (Fig. 3b). Then, each experiment is evaluated singularly and after all runs are completed, the best point of the first iteration is chosen in terms of highest STY (Fig. 3c). In the second run, the reaction is further optimized by investigating the experimental space near the best point found in the first iteration, which was taken as center point (Fig. 3d). In this phase, the geometrical size of the CCD was set to 40 % of the first iteration, therefore ensuring that no point would lie outside the initial boundaries of the design space. The overall amount of experiments needed is 21, of which 11 were carried out in the first run and 10 in the second. The combined size of the two iterations ensured, that 98 % of the reaction space can be explored via this method.

The DoE was executed via the automated reaction platform shown in Scheme 3. A Python-based algorithm with an integrated graphic user interface (GUI) was developed to automatically choose the DoE points, according to the experimental space limits defined by the user, and to set the thermostat and the pumps at the desired level for each experimental run. The same algorithm saved and accessed the UV-Vis spectra recorded by the spectrometer in order to calculate the compound concentrations, yield and STY of each experiment. These variables were chosen as response parameters to assess the result of each experiment and determine the optimum combination of factors. The final output parameter that was to be maximized via the DoE was STY, as this parameter can take into account not only the yield of the reaction but also the influence of flowrate and free volume in the column. Once all of the experiments were performed on one column, the algorithm prompted the six-way-valve to switch the feed to the second column, onto which the DoE approach was repeated as before. After having evaluated both inserts, the algorithm returns the results of the DoE, and chooses the best insert and set of process parameters. The columns could then be manually removed from the setup and the inserts exchanged to the other design type for further experiments, which were carried out as explained above.

**Fig. 4:**
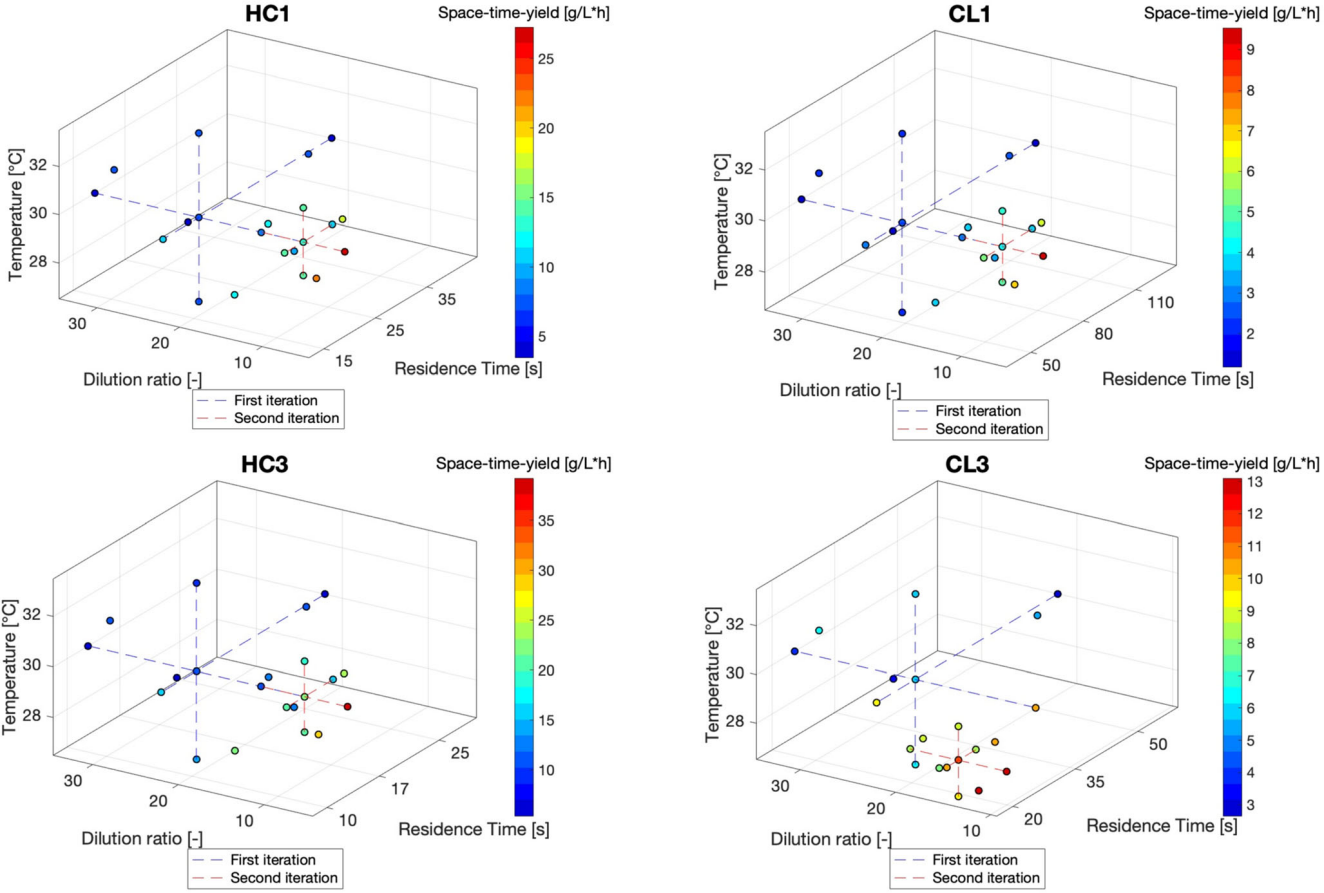
Graphical summary of the DoE results for the 4 structured inserts investigated. The cross points for the first iteration are connected via blue dashed lines, while for the second iteration they are connected in red. The color map to the right of each graph gives an indication of the level of STY reached for each experimental point. The results are plotted in terms of Residence time rather than flowrate, as the first differs among each insert depending on the internal volume. The best point in the first iteration for HC1, HC3 and CL1 was 30 °C,1.1 mL/min and a dilution of 9.4. For CL3 instead it was 28 °C, 1.464 mL/min and 14.71 dilution. For the second iteration, the best combination of factors was 30 °C, 1.1 mL/min and a dilution ratio of 4.36 for HC1, HC3 and CL1. For CL3 the best point was 28 °C, 1.464 mL/min and ad dilution ratio of 9.676

The results for the optimization experiments are plotted in Fig. 4 and reported in Table 4. At the end of the second iteration, the best combination of factors for HC1, HC3 and CL1 was 30 °C, 1.1 mL/min and a dilution ratio of 4.36, which corresponds to a starting concentration of 1.86 mM. For these inserts, it was apparent that the optimum temperature lays around 30 °C, which is in line with what was already suggested in previous works [34, 48]. Moreover, for all of them a combination of a medium flowrate and lowest dilution ratio was preferred, as this gives the highest productivity, thereby optimizing the STY. The picture is instead different for CL3; for this insert the best point was 28 °C, 1.464 ml/min and dilution ratio of 9.676 which corresponds to a starting concentration of 0.93 mM. So in this case the algorithm went down another path for optimization, but still preferred a compromise between flowrate and dilution ratio to increase productivity. The reason for which in this case a different optimum was found is clear when looking at Fig. 4 (CL3): in the first iteration, the points at 28 °C,1.464 mL/min and 0.63 mM (dilution ratio of 14.71) and at 30 °C, 1.1 mL/min and 0.96 mM (dilution ratio of 9.4) resulted in a very close STY, but since the algorithm goes for the best point and does not explore the space around the second best, the second iteration went in a different direction. A more complex optimization algorithm could identify and evaluate also different local optima, however for the purpose of this work the chosen DoE approach was enough to identify a trend to optimize the overall productivity.

**Table 4:** Summary of the DoE results for each structured insert used in this work

	First iteration		Second iteration		
Type of insert	Yield [%]	STY [g/L·h]	Yield [%]	STY [g/L·h]	Residence time [s]
HC1	62.6	13.59	64.7	27.27	19.15
HC3	70.9	23.11	62.2	39.21	12.8
CL1	59.2	4.32	67.3	9.53	56.95
CL3	77.6	11.16	61.9	13.11	19.14

The STY achieved at steady state for the best combination of parameters in each insert is summarized in Table 4. For all the investigated inserts, the final STY was higher than the value of 4.8 g/L·h ,which was achieved in a previous work [34]. As shown in Table 4, HC3 gave the best results in terms of STY: at the optimum conditions, a value of 39.21 g/L·h was reached, which represents a 8-fold increase compared to what was achieved previously. This result can be explained by the fact that HC3 has the lowest internal volume and the highest surface to volume ratio, thus the highest enzyme coverage. Therefore, HC3 was deemed as the best choice to perform the decarboxylation of coumaric acid in continuous flow, as it results in a higher amount of substrate being converted in time and also offered the best mixing properties compared to all designed inserts. Moreover, compared to the previous work, the application of an automated flow setup together with a systematic DoE strategy proved to be a successful approach to rapidly screen the effect of different parameters on the outcome of a reaction by minimizing the need of human intervention and the time needed for evaluation. Also, the reaction could be followed in real time via implementing an online measurement, which is more reliable compared to offline methods and does not require sample handling/manipulation [60], thus saving time and resources. Finally, the possibility of easily designing and manufacturing interchangeable inserts via means of 3D printing, allows to rapidly adapt to the reaction needs to achieve optimal process conditions. These advantages, coupled with the improved productivity, represent definitely a step forward towards a more industrially feasible enzymatic conversion of coumaric acid to vinylphenol.

## Conclusions

In conclusion, we have proved in this work how 3D printing can be used as a powerful tool to design and produce advanced pieces of equipment for many applications in flow. First, novel 3D printed ceramic structured inserts have been designed and 3D printed via VPP. In order to characterize the fluidic behavior inside the inserts, RTD measurements were carried out with the aid of self-made 3D printed photometric flow cells. These tools were used to determine the exit age distribution for each insert via recording the step input of a tracer at different operating conditions. By recording the tracer input at both the inlet and the outlet of a reactor, it was possible to remove any disturbances caused by the feeding capillary, thereby increasing the quality of the results and proving to be a valid low-cost alternative to expensive inline analytical equipment. The evaluation of the RTD results further allowed to conclude that all inserts approach the fluidic behavior of theoretical CSTR reactors. Then, as an application-oriented proof of concept, the inserts were enclosed in HPLC columns to be used as solid supports onto which *bs*PAD was immobilized for the catalytic conversion of coumaric acid to vinylphenol in continuous flow. To identify the optimal conditions for the selected system, a fractional factorial CCD was chosen as a DoE approach to systematically investigate the effect of different process parameters (temperature, flowrate and dilution ratio) on the STY. The DoE experiments were successfully carried out in an automated platform controlled by a Python-based algorithm, which automatically chose the DoE points based on design space given by the user and run the experiments by remotely controlling the equipment, reducing the need for human intervention and saving time and materials. As a result, the HC3 insert proved to be the best choice for the production of vinylphenol, as it gave a STY of 39.21 g/L·h, representing an 8-fold increase compared to the value of 4.8 g/L·h obtained previously. These results showed that it is possible to combine different advantages of flow chemistry and 3D printing to biocatalytic processes, with great results in terms of productivity. In the near future, the presented approach combining 3D printing and process automation will be applied to further enzymatic reaction systems, in order to increase the feasibility of biocatalysts for their application in industrial processes. Moreover, the presented inserts will also be implemented as supports for other active species and tested in different catalytic applications.

## Supplementary Information

ESM 1 (PDF 1.55 MB)

## Abbreviations

API: Active Pharmaceutical Ingredient
BET: Brunauer–Emmett–Teller
*Bo*: Bodenstein number
*bs*PAD: Phenolic acid decarboxylase from *Bacillus Subtilis*
CAD: Computer Aided Design
CSTR: Continuous Stirred Tank Reactor
DES: Deep Eutectic Solvent
DoE: Design of Experiments
CCD: Central Composite Design
CL: Cubic Lattice
GUI: Graphical User Interface
HC: Honeycomb
ID: internal diameter
ME: material extrusion
OD: outer diameter
PFR: Plug Flow Reactor
*Re*: Reynolds number
RTD: Residence Time Distribution
SLA: Stereolithography
STY: Space-Time-Yield
VPP: vat photopolymerization
