# Supplementary material for: 3D printed ceramics as solid supports for enzyme immobilization: an automated DoE approach for applications in continuous flow
Source: J Flow Chem. 2021 Apr 29;11(3):675–89. doi: 10.1007/s41981-021-00163-4 (PMC8563604; doi:10.1007/s41981-021-00163-4)
Supplement: Supplementary file 1 — (PDF 1.55 MB) [file 41981_2021_163_MOESM1_ESM.pdf]

Supplementary Material

Content

General **.......... *2***

1. Designing and printing of the supports **.......... *2***

1.1 Building parameters 3

2. Residence time distribution **.......... *3***

2.1 Design and printing of the RTD flow cells and measurement circuit 3

2.2 RTD experiments – Standard operating procedure 5

2.3 RTD experiments – E curves 5

3. Production and immobilization of bsPAD **.......... *7***

3.1 Enzyme expression and lyophilization 7

3.2 Investigated immobilization procedures 7

3.3 Preliminary batch experiments – Activity assays 10

4. Analytics and equipment **.......... *11***

4.1 HPLC measurement 11

4.2 UV-Vis spectrophotometer 12

4.3 Specific surface area measurement 12

References **.......... *13***

General

All chemicals and solvent were purchased from Sigma Aldrich and TCI Chemicals unless stated otherwise and used as received.

1. Designing and printing of the supports

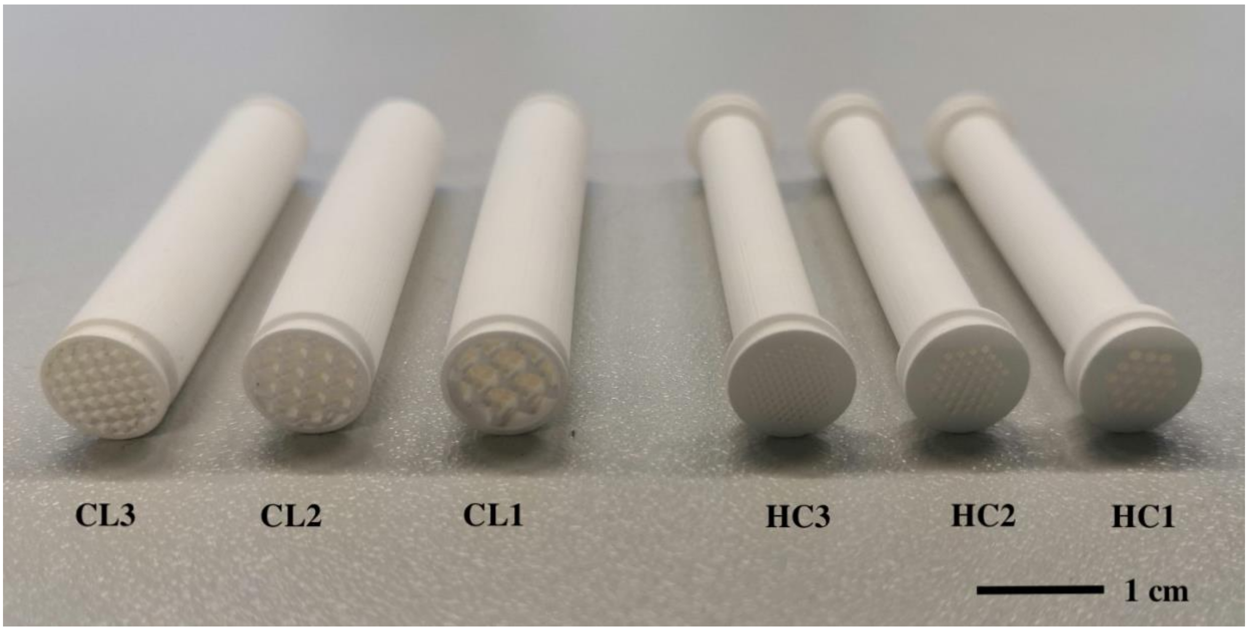

Figure S1- All the designed and investigated 3D printed supports, after being manufactured by VPP. CL stands for Cubic lattice, while HC for Honeycomb.

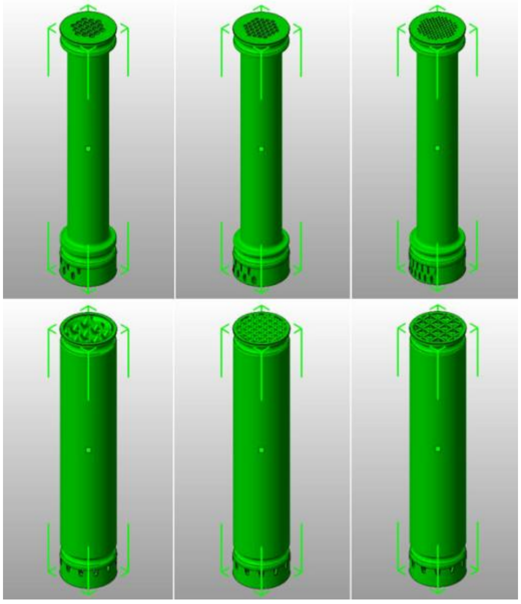

Figure S2 – Rendered image of the 3D printed supports in the printer software prior to fabrication. Replicated with permission from Lithoz GmbH.

The design and fabrication of the inserts was carried out in an iterative approach, where the inserts were initially printed with commercially available resins. This phase allowed to visualize the designs and perform preliminary tests on them. Also in this phase the manufacturing limits and parameters were defined (e.g. minimum printable channel size) before proceeding with the ceramics printing.

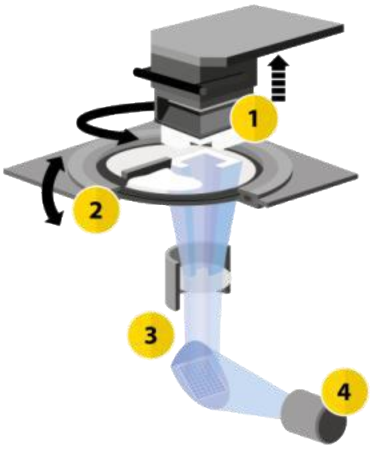

Figure S3 - Schematic representation of the lithography-based printer CeraFab 7500 used to fabricate the structured inserts presented in this work. Reproduced with permission from Lithoz GmbH.

1.1 Building parameters

Machine preparation	
Temperature [°C]	25
**Job parameters**	
Geometry	
Layer thickness [µm]	25
Total amount of layers	2050
Time per layer [s]	60
Geometry corrections	
Lateral (XY) shrinking compensation	1.125
Build direction (Z) shrinking compensation	1.250
Exposure	
DLP energy general [mJ/cm2]	470

Table S1 – Summary of the building parameters used for the fabrication of the structured inserts presented in this work.

2. Residence time distribution

2.1 Design and printing of the RTD flow cells and measurement circuit

A 3D-printable flow cell (Figure S4) was designed to be mountable before and after a reactor. It is directly attachable to a commercial 1/16” capillary and hold in place by 1/4”-28 flat bottom fittings. The photometric measurement is enabled by a light source, which gets directly squeezed into one of the ports and a photo resistor placed on the mounting plug. Emitted light of a red laser LED shines through the capillary and is recognized by a changing resistance of a photo resistor. The path length of the measurement is determined only by the used internal diameter of the capillary, because additional material was added to only allow light to pass through the capillary. The flow cell was printed with an Anycubic Mega S from Anycubic with a layer height of 0.2mm.

An Arduino Nano was used to continuously transfer the internally calculated absorption of each flow cell to a PC and is soldered with all other components on a PCB. Two buttons are included in the design for turning the LEDs on and off as well as taking a reference light and dark spectra for the calculation of absorbance. The changing voltage of this optical measurement is recognized by an ADS1115. This chip was used since its resolution of 16bit and the possibility of a programmable gain amplifier showed a better performance compared to the 10bit analogue digital converter of the Nano. Additionally, a voltage divider with a 10kΩ potentiometer was used as power supply for the photo resistor. The voltage divider allows to change the offset of each flow cell.

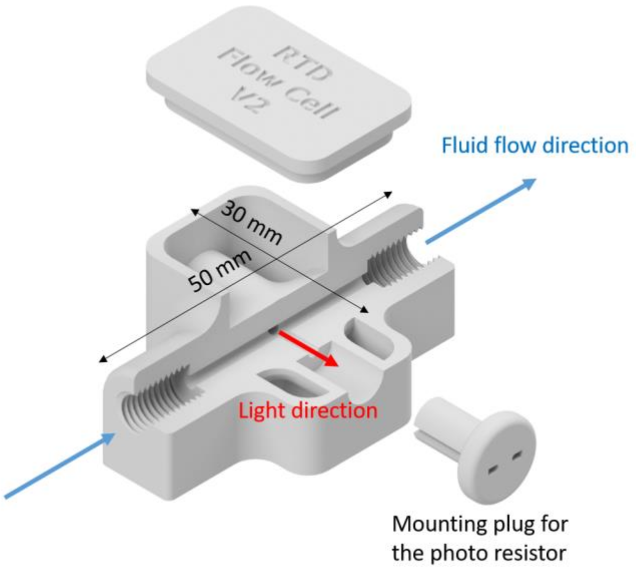

Figure S4 – CAD drawing for the 3D printed inline spectroscopy flow cells. They are equipped with a case into which both a laser light source and a photoresistor can be inserted. The flow through the flow cell is made possible by connecting 1/16” PTFE capillaries via standard HPLC flat bottom fittings.

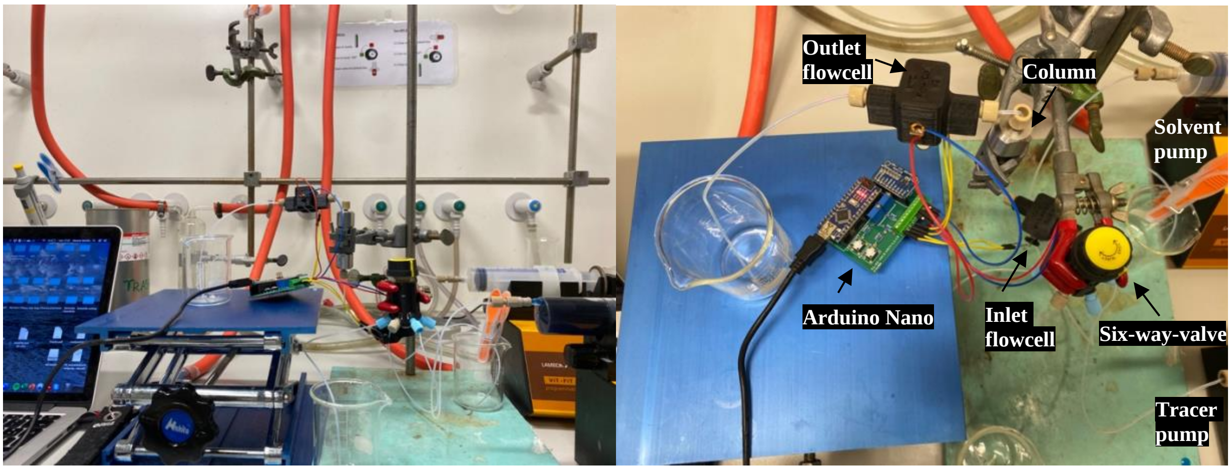

Figure S5 – Experimental setup for the RTD experiments: view from the front (left); view from the top (right), with indication of the important elements of the setup.

2.2 RTD experiments – Standard operating procedure

The experimental setup was assembled as shown in the main article. It comprises of two syringe pumps, one that delivers solvent and one tracer solution. Both are connected to a six-way-valve, to which also the microfluidic device is attached. In the case of the 3D printed catalytic inserts presented in this work, they were fitted in commercially available HPLC columns, in order to constitute the structured bed reactors to be studied. Two 3D printed spectroscopy flow cells were also installed, one at the inlet and one at the outlet of the column. The connections within the setup are made via PTFE capillaries (1/16" OD, 0.03" ID) and standard HPLC fittings. The distance between the flow cells and the column was kept as short as possible to reduce falsification of the results.
For the preparation of the tracer solution, 12 mg of methylene blue were dissolved in 100 mL 96 v% ethanol. The amount of methylene blue was chosen to give an appreciably high absorbance (around 0.22) that can be measured during the experiment. The prepared tracer solution was filled in a 50 ml syringe. A second 50 ml syringe is filled with 96 vol.% ethanol, and both syringes were each installed on syringe pumps and connected to the six-way-valve. The pumps were connected in a way that while in the valve is in the “Load” position, only solvent was pumping through the setup, while by switching the six-way-valve to “Inject” caused a step input of tracer to be injected in the setup.
Before starting the experiment, the Arduino Nano was connected to the computer via USB. The LED lamp of each flow cell was switched on by pressing the LED-button on the circuit and let warm up for about 10 minutes to ensure steady operating conditions. The program Tera Term is used as a serial data logger on the computer to log the experiment and save the raw data as .log file.
After having set up Tera Term, the first step was to flush the whole setup with only ethanol. This preliminary flushing phase was used to take a dark reference for both flow cells by switching off the LED and pressing the reference button on the Arduino Nano. After this the LED lamps were switched on and the reference button was pressed again to collect a light reference.
Before an experiment, the flow rate of each syringe pump was adjusted to the desired value (both pumps were set to the same flow rate). To start the experiment, first the syringe pump with the tracer was switched on to fill up the connection capillaries before the injection. Once the capillaries were filled, the six-way-valve was turned to the inject position and the measurement of the step input was started by recording the absorbance detected by both flow cells. The step-up measurement was finished after the measured absorbance at both flow cells reached a stable value for at least one mean residence time of the column. After this time had passed, the solvent pump was switched on and the six-way-valve was turned to the load position and the system was flushed with clean solvent. When the value of the measured absorption of both flow cells reached 0.00, the step-down measurement was finished and the pumps could be adjusted to a new setpoint. If the value of the absorption did not reach the desired 0.00 after several minutes, the flow cells were referenced again, as the baseline could have shifted due to changes in the system.
After all operating points had been evaluated, both pumps were switched off and the Tera Term program was closed. Then the .log file of the experiment was exported in a Microsoft Excel sheet for further interpretation of the obtained raw data.

2.3 RTD experiments – E curves

The exit age distributions (E) calculated for all structured inserts with respect to the dimensionless time θ are reported in Figure S6. For all the inserts, a fluidic behavior very far from plug-flow was measured at all investigated flowrates, as visible from the irregular shape of the E curves. The measurements, especially for lower flowrates and bigger channel diameters, resulted to be noisy and affected by disturbances in the flow. These disturbances could be attributed to a number of

sources. First of all, at low flowrates the extent of backmixing in the devices is very high, resulting in an irregular fluidic pattern which is mirrored by the irregular shape of the E curve. Secondly, at low flowrates the syringe pumps used start to show a pulsating behavior, which causes the tracer to not be injected with an ideal step. Also, being self-made, the RTD flow cells were more sensitive to disturbances compared to more sophisticated spectroscopy equipment, however they can be used for a quick but still reliable estimation of the fluidic properties inside the inserts.
Moreover, the presence of strong internal recirculation is indicated by the small decaying peaks noticeable for all of the E curves[1]. Also, small initial peaks were detected, which could derive from channeling inside the inserts and flow maldistributions. In fact, the inlet capillary and the column are tied via a connector with a small inlet in the middle, which might cause the fluid to travel only through the central channels and not get equally distributed among all of them, especially at lower flowrates.
Reasonably slim and symmetric curves could be obtained for higher flowrates (2 and 1 ml/min) and for the HC3 and CL3 support, as these showed the lowest extent of backmixing due to smaller internal diameter. The reason behind it is that at higher flowrates and small diameter of the channels, the fluid is forced into the outer channels due to a higher backpressure of the channels in the middle. In this way, the fluid is better distributed along the channels and the axial dispersion is reduced. The lower the flowrate, the more irregular are the E curves, resulting in higher axial dispersion and deviation from the plug-flow fluidic pattern.

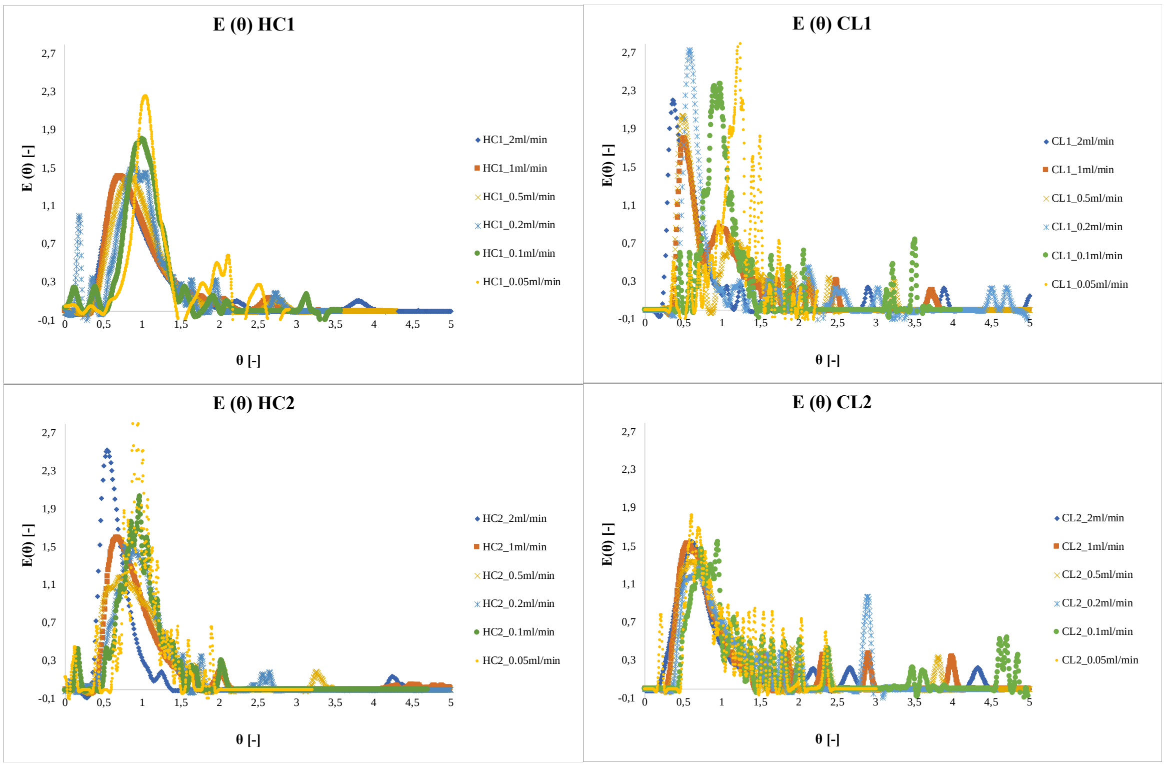

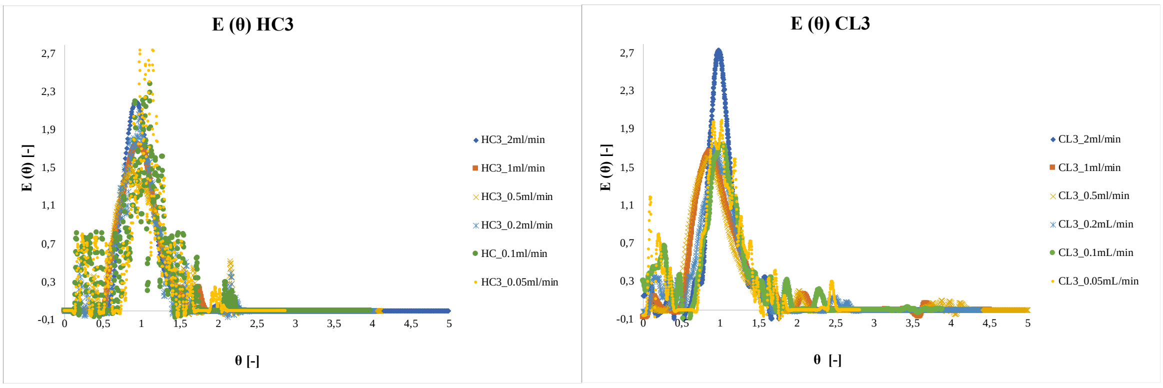

Figure S6 – Exit age distribution curves for all the structured reactors studied in this work.

3. Production and immobilization of *bs*PAD

3.1 Enzyme expression and lyophilization

The enzyme was produced at the Institute for Molecular Biotechnology, TU Graz, as already described in a previous work[2]. The recombinant pET28a expression plasmid, containing the padC gene (Gene ID: 398579) encoding for PAD from Bacillus subtilis, was constructed as described elsewhere[3]. Chemo-competent E. coli BL21 (DE3) cells were transformed with the expression plasmid and single colonies were used to inoculate overnight cultures (5 mL LB-Kan, 40 µg/µL kanamycin), which were incubated at 37 °C and 130 rpm. The complete overnight culture was used to inoculate 200 mL TB-Kan medium in 1 L baffled flasks. Cultures were incubated at 37 °C and 130 rpm, until OD600 reached 0.5-0.7 and protein expression was induced by addition of IPTG to a final concentration of 0.1 mM. After incubation at 20 °C and 120 rpm for 20-24 h the cells were harvested by centrifugation (15 min, 4500 rpm, 4 °C) and the cell pellet was washed once with 50 mM KPi buffer (pH 6). Cells were either stored at -20 °C or directly used for the preparation of cell-free extract. For the preparation of the lyophilized cell free extract, cell pellets were resuspended in 50 mM KPi buffer (pH 6) to a concentration of 100 mg_{CWW} mL-1 and lysed by sonication (Branson sonifier 250; 5 min, Duty cycle 5, Output control 50 %). The cell-free extract obtained after centrifugation (20 min, 11000 rpm, 4 °C) was sterilized by filtration, shock-frozen in liquid nitrogen and directly used for freeze-drying (AdVantage Pro Lyophilizer, SP Scientific). The lyophilized cell-free extract was stored at -20 °C until further use.

3.2 Investigated immobilization procedures

Two immobilization procedures were tested onto ceramic particles produced by crushing and sieving the structured inserts that failed during the printing phase.

The first procedure (indicated as Method 1) was taken and slightly modified from literature[4]. In order to activate the carrier material's surface, this was kept in contact with 37% HCl for 30 min at room temperature. Then it was washed extensively with water until the pH of the washing solution was neutral. The second step involved the silanization of the surface using APTES as a reagent. The support was kept in contact with an APTES solution of 2,5% in deionized water (pH 7) at 80°C for 3 hours. The carrier was extensively washed with water and let cool down to room temperature. In order to introduce aldehyde functional groups, the APTES layer was let react with a solution of 5% glutaraldehyde in the optimal buffer identified for *bs*PAD (50 mM phosphate buffer pH 6) for one hour. After the reaction was complete, the originally white ceramic material showed a slight pink coloration. As a final enzyme immobilization step, the support was placed in a solution of 16 mg/mL of *bs*PAD CFE in KPi buffer pH for 20 hours at room temperature. If not used right away, the carrier was stored in a falcon tube filled with KPi buffer at 4°C.

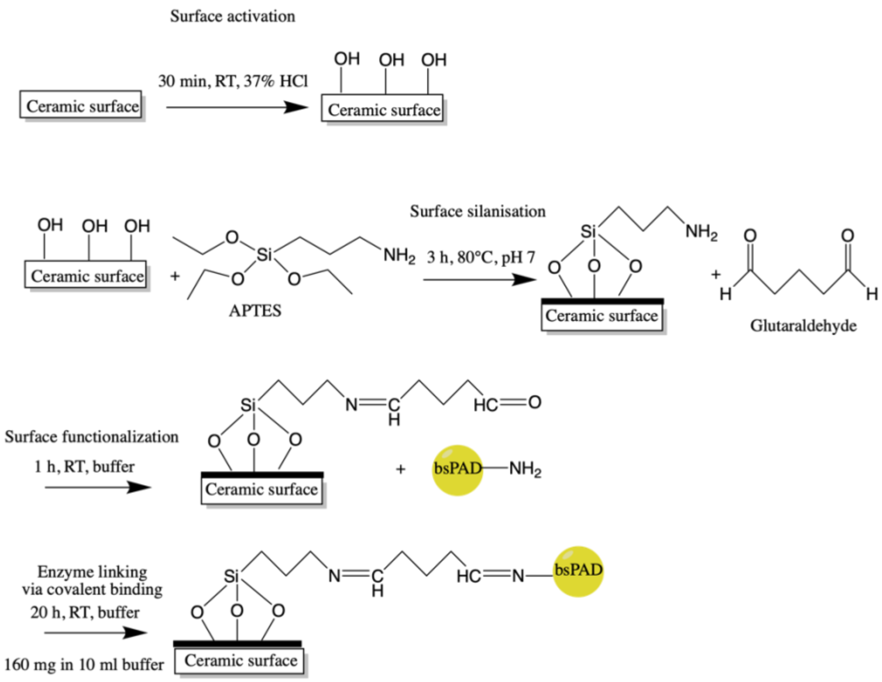

Figure S7 – Scheme of the immobilization steps for the first immobilization protocol, adapted from literature[4].

The second procedure (Method 2) was taken with slight modifications from literature[5,6]. The surface activation and silanization steps were the same as in the first protocol. This time, enzyme linking was induced using EDC hydrochloride to activate the carboxylic group available on the enzyme and NHS as a linking agent to the silanized support surface. This was achieved by using a 10:1 weight ratio of NHS plus 5:1 weight ratio of EDC (with respect to the CFE) in KPi buffer together with enzyme solution (16 mg/mL *bs*PAD CFE in the same buffer). The supports were kept in this solution for 20 hours at room temperature.

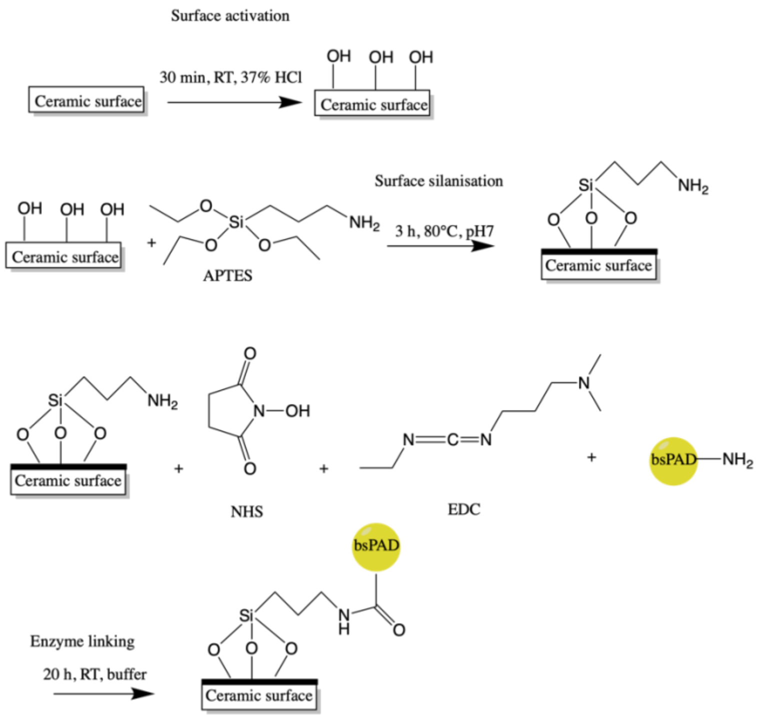

Figure S8 – Scheme of the immobilization steps for the second immobilization procedure, as adapted from literature[5,6].

All the steps for immobilization involving ceramic particles as carriers were conducted in batch. The particles were charged into a round bottom flasks together with the required solution (according to the immobilization step), then they were filtered and washed after each step, which increased particle loss and the time needed for immobilization. For the structured inserts, the immobilization was instead conducted in flow, in a setup shown in Figure S9. The insets were fitted inside HPLC columns and connected in parallel. The setup involved a syringe pump, which was connected to the columns via standard HPLC fittings. A back pressure regulator (BPR) was attached at the outlet to apply a uniform pressure on the system and allow a homogeneous distribution of the different immobilization solutions in the inserts' channels. The immobilization procedure was generally carried out by connecting 2 columns of the same type at a time, in order to reduce flow maldistributions. By using this setup, it was possible to carry out the different steps of the immobilization by simply switching the syringe and filling it with the required solution. This is one of the advantages of using such structured inserts, as the intermediate filtration/filling/discharging steps are not needed and the overall immobilization procedure is faster.

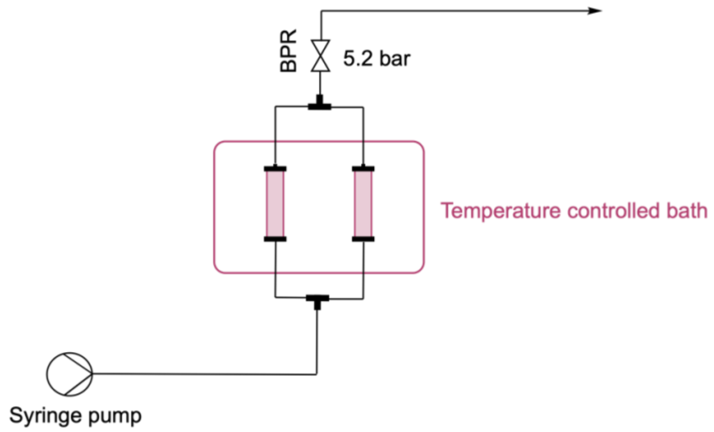

Figure S9 – Graphical representation of the setup used of the continuous immobilization of *bs*PAD on the structured inserts.

3.3 Preliminary batch experiments – Activity assays

The immobilization of *bs*PAD was first tested onto particles obtained by crushing and sieving the structures that failed during the printing phase. The purpose of these preliminary tests was to study the activity of *bs*PAD immobilized with the two different methods described in the previous section. The results are reported in Table S2 and plotted in Figure S10.
1 g of crushed ceramic powder was suspended in the solvent mixture of 2.5 mL DES (choline chloride/glycerol 1:2 (mol/mol)) and 2.5 mL potassium phosphate buffer (50 mM, pH 6.0) in a 10 mL round bottom flask and heated to 30 °C. 57.5 mg (0.7 mmol) of para-coumaric acid were added and samples were taken after 0, 15, 30, 60, 120 and 240 min. For each sample 50 µL of the reaction mixture were diluted with 500 mL of HPLC eluent (methanol: HPLC buffer 7:3, HPLC buffer = water:H_3PO_4 300:1, see Section 4.1 for the specific method used).
The outcome of the immobilization on the support particles was determined by calculating the immobilization yield by taking 1 mL of the starting and ending immobilization solutions and performing the activity assay as described above. The immobilization yield and the activity were calculated as shown in the main article. The activity of the enzyme is given in units U [µmol/min], and was determined directly for *bs*PAD immobilized onto the ceramic particles from the results of the batch experiments. The residual activity A_R on the ceramic particles was also calculated by repeating the batch experiments after a week, and was determined as percentage of catalytic activity left after one week at storage conditions (4°C, KPi buffer 50 mM pH6).

	After immobilization			After 1 week		
	**dc/dt [µmol/ml*min]**	**U [µmol/min]**	**Y_i [%]**	**dc/dt [µmol/ml*min]**	**U [µmol/min]**	**A_R [%]**
Method 1	0.177	0.885	18.46	0.141	0.702	79
Method 2	0.269	1.345	42.21	0.267	1.336	99

Table S2 – Summary of the results for the preliminary *bs*PAD immobilization tests conducted onto crushed ceramic powder.

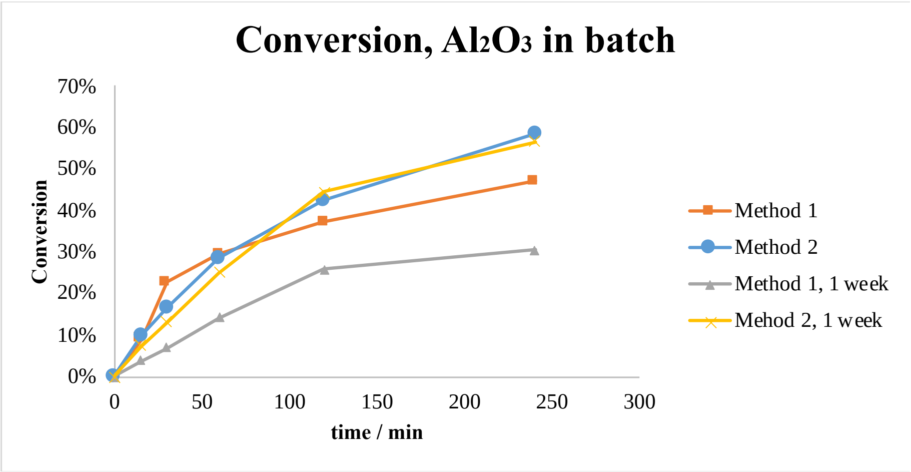

Figure S10 – Conversion of coumaric acid into vinylphenol versus reaction time for different reaction tests onto crushed ceramic powder.

Results showed that the enzyme immobilized with method 2 displayed greater activity compared to method 1, as well as higher long-term stability. Therefore, method 2 was chosen for the immobilization of *bs*PAD onto the structured inserts.

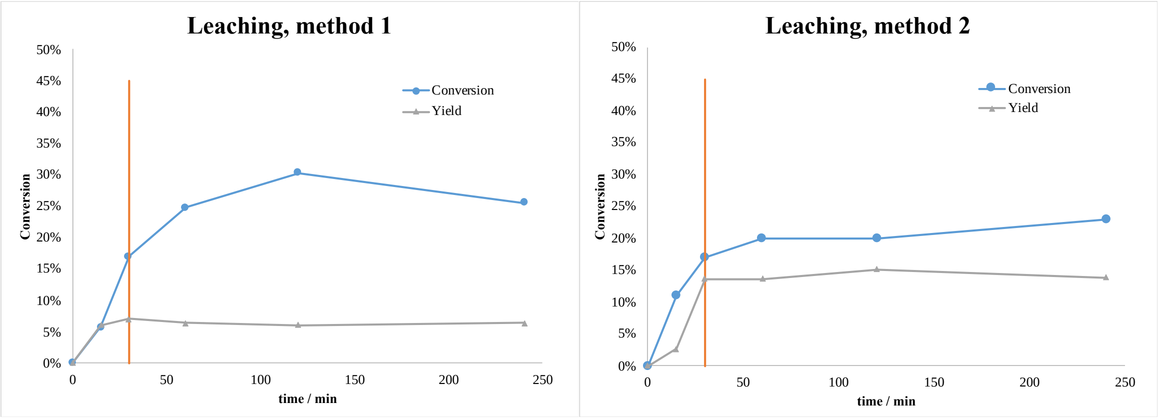

Figure S11 – Results from the leaching studies for *bs*PAD immobilized on the ceramic particles with the two different immobilization procedures. The orange straight line indicated the point at which the particles were filtered out.

Leaching studies were also carried out to compare the two procedures even further. In these studies, the batch reaction was set up as described above, but the particles were filtered out of the reaction solution after the first 30 minutes. Then, the remaining solution was kept stirring at 30°C and samples were taken at 60, 120 and 240 minutes and measured with HPLC. As shown in Figure S11, the yield of vinylphenol stayed constant in both cases. However, the concentration of coumaric acid was fluctuating, resulting in an unstable value of the conversion. This result was attributed to the poor solubility of coumaric acid in aqueous environments, or its possible adsorption on the ceramic surface.

4. Analytics and equipment

4.1 HPLC measurement

In order to monitor the reaction progress during the batch experiments, reaction samples were taken and analyzed using an Agilent 1100 series HPLC system equipped with an online degasser, quaternary pump, autosampler, thermostated column compartment and UV-visible diode array detector. As mobile phases, Buffer (A, H_2O:H_3PO_4 = 300:1, v/v, Sigma Aldrich, Darmstadt, Germany) and HPLC grade methanol (B; Chemlab, Zedelgem, Belgium) were used. Compounds were separated using a ThermoFischer Scientific Accucore^{TM} C18 reversed phase column (50 x 4.6 mm; 2.6 µm) at 25 °C with a flow rate of 1 mL/min and detected by UV-absorption over the run time of 23 min. The injected volume for each sample was 2µL. The used elution method is summarized in Table S3.
A calibration curve was determined for both coumaric acid and vinylphenol, with 3 points and $R^2 \geq 0.99$. Calibration slopes [area/mM]: coumaric acid, 1012 (measured at 282 nm); vinylphenol, 375 (measured at 237 nm).

Table S3 HPLC method (% A = % H_2O:H_3PO_4=300:1 v/v, % B = % MeOH) for monitoring of the reaction progress.

Time [min]	% A (v/v)	% B (v/v)	Sample diluent	Flow [mL/min]
0-1	60	40	MeOH:Buffer=40:60	1
1-12	10	90		
12-14	60	40		
14-23	80	20		

4.2 UV-Vis spectrophotometer

An Avantes AvaLight-DS-DUV, equipped with a deuterium lamp, was used as light source for UV-Vis measurements and an Avantes AvaSpec-ULS2048 was used as detector. They were connected with two optical fibers FC-UV400-1-FIA-SR to an Avantes Micro Flow Cell with 1.5 mm optical path length.
A calibration curve was determined for both coumaric acid and vinylphenol, with 3 points and $R^2 \geq 0.99$. Calibration slopes [absorbance/mM]: coumaric acid, 1.812 (measured at 325 nm), 0.728 (measured at 260 nm); vinylphenol, 0.446 (measured at 260 nm).

4.3 Specific surface area measurement

Specific surface areas for each uncoated support were measured using a Tristar II 3020 (Micromeritics, Norcross, Georgia) with nitrogen as analytical gas. The supports were degassed under vacuum at ambient temperature for 24 h. The volume of nitrogen adsorption was recorded over a relative pressure range between 0.01 and 0.99. 15 points in the relative pressure range of 0.01–0.2 were used for the calculation of the surface area according to the Brunauer-Emmet-Teller (BET) theory. The pore width and diameter were calculated by the Barrett−Joyner−Halenda (BJH) method. The measured properties for each insert are summarized in Table S4.

**Type**	**BET area** [m^2/g]	**Pore volume** [cm^3/g]	**Pore diameter** [nm]	**Weight** [g]	**Area** [m^2]
HC1	0.254	0.000507	8.717	2.314	0.587
HC2	0.310	0.00062	8.177	2.498	0.774
HC3	0.543	0.00174	13.074	2.858	1.551
CL1	0.484	0.00154	12.636	1.987	0.962
CL2	0522	0.00143	11.004	2.519	1.305
CL3	0.536	0.00152	12.412	3.766	2.017

Table S4 – Summary of the surface characteristic determined via BET measurement for all the designed 3D printed inserts.

References

(1) Levenspiel, O. *Chemical Reaction Engineering*, 3rd ed.; John Wiley & Sons: New York, 1999.

(2) Grabner, B.; Schweiger, A. K.; Gavric, K.; Kourist, R.; Gruber-Woelfler, H. A Chemo-Enzymatic Tandem Reaction in a Mixture of Deep Eutectic Solvent and Water in Continuous Flow. *React. Chem. Eng.* **2020**, *5* (2), 263–269. https://doi.org/10.1039/c9re00467j.

(3) Gómez Baraibar, Á.; Reichert, D.; Mügge, C.; Seger, S.; Gröger, H.; Kourist, R. A One-Pot Cascade Reaction Combining an Encapsulated Decarboxylase with a Metathesis Catalyst for the Synthesis of Bio-Based Antioxidants. *Angew. Chemie - Int. Ed.* **2016**, *55* (47), 14823–14827. https://doi.org/10.1002/anie.201607777.

(4) Thomsen, M. S.; Nidetzky, B. Coated-Wall Microreactor for Continuous Biocatalytic Transformations Using Immobilized Enzymes. *Biotechnol. J.* **2009**, *4* (1), 98–107. https://doi.org/10.1002/biot.200800051.

(5) Hollermann, G.; Dhekane, R.; Kroll, S.; Rezwan, K. Functionalized Porous Ceramic Microbeads as Carriers in Enzymatic Tandem Systems. *Biochem. Eng. J.* **2017**, *126*, 30–39. https://doi.org/10.1016/j.bej.2017.06.015.

(6) Halfer, T.; Rei, A.; Ciacchi, L. C.; Treccani, L.; Rezwan, K. Selective Covalent Immobilization of Ferritin on Alumina. *Biointerphases* **2014**, *9* (3), 031018. https://doi.org/10.1116/1.4895688.
